# High *Frankia* abundance and low diversity of microbial community are associated with nodulation specificity and stability of sea buckthorn root nodule

**DOI:** 10.3389/fpls.2024.1301447

**Published:** 2024-02-21

**Authors:** Hong Liu, Bingbing Ni, Aiguo Duan, Caiyun He, Jianguo Zhang

**Affiliations:** ^1^ State Key Laboratory of Tree Genetics and Breeding & Key Laboratory of Tree Breeding and Cultivation, National Forestry and Grassland Administration, Research Institute of Forestry, Chinese Academy of Forestry, Beijing, China; ^2^ Collaborative Innovation Center of Sustainable Forestry in Southern China, Nanjing Forestry University, Nanjing, China

**Keywords:** sea buckthorn, nodule, metagenome, environmental factors, *Frankia* sp. EAN1pec

## Abstract

**Introduction:**

Actinorhizal symbioses are gaining attention due to the importance of symbiotic nitrogen fixation in sustainable agriculture. Sea buckthorn (*Hippophae* L.) is an important actinorhizal plant, yet research on the microbial community and nitrogen cycling in its nodules is limited. In addition, the influence of environmental differences on the microbial community of sea buckthorn nodules and whether there is a single nitrogen-fixing actinomycete species in the nodules are still unknown.

**Methods:**

We investigated the diversity, community composition, network associations and nitrogen cycling pathways of the microbial communities in the root nodule (RN), nodule surface soil (NS), and bulk soil (BS) of Mongolian sea buckthorn distributed under three distinct ecological conditions in northern China using 16S rRNA gene and metagenomic sequencing. Combined with the data of environmental factors, the effects of environmental differences on different sample types were analyzed.

**Results:**

The results showed that plants exerted a clear selective filtering effect on microbiota, resulting in a significant reduction in microbial community diversity and network complexity from BS to NS to RN. Proteobacteria was the most abundant phylum in the microbiomes of BS and NS. While RN was primarily dominated by Actinobacteria, with *Frankia* sp. EAN1pec serving as the most dominant species. Correlation analysis indicated that the host determined the microbial community composition in RN, independent of the ecological and geographical environmental changes of the sea buckthorn plantations. Nitrogen cycle pathway analyses showed that RN microbial community primarily functions in nitrogen fixation, and *Frankia* sp. EAN1pec was a major contributor to nitrogen fixation genes in RN.

**Discussion:**

This study provides valuable insights into the effects of eco-geographical environment on the microbial communities of sea buckthorn RN. These findings further prove that the nodulation specificity and stability of sea buckthorn root and Frankia sp. EAN1pec may be the result of their long-term co-evolution.

## 1 Introduction

Although nitrogen is abundant in the atmosphere, plants cannot use it directly. Therefore, nitrogen is usually one of the most limiting nutrients for plant growth and development (LeBauer and Treseder, 2008). The acquisition of nitrogen in nature by plants is entirely reliant on fixed nitrogen, making nitrogen fixation a critical process for the stability and sustainability of ecosystems (Roy et al., 2019). Studies have shown that root nodule symbiosis (RNS), which encompasses legume-rhizobial and actinorhizal symbioses, is the most efficient nitrogen fixation process (Ardley and Sprent, 2021). Currently, most of the rhizobial population belongs to genera of the alpha-Proteobacteria class, including (*Rhizobium, Bradyrhizobium, Sinorhizobium, Ensifer, Mesorhizobium, Agrobacterium, Azorhizobium, Allorhizobium, Shinella, Devosia, Neorhizobium, Pararhizobium, Phyllobacterium, Microvirga, Ochrobactrum, Methylobacterium*) and beta-Proteobacteria (B*urkholderia, Cupriavidus, Paraburkholderia, Trinickia*) (Estrada-de los Santos et al., 2018; Kuzmanović et al., 2022; Rajkumari et al., 2022). Moreover, some gamma-Proteobacteria (*Pseudomonas*) has also been reported to form effective nodules, but *Pseudomonas* are not traditionally rhizobial (Shiraishi et al., 2010). An increasing number of studies have shown that rhizobia species and abundance in legume nodules are influenced by geographical environmental characteristics, plant species and genotypes, and soil physicochemical characteristics (Xiao et al., 2017; Stefan et al., 2018; Zhang et al., 2018a; Sharaf et al., 2019; Hakim et al., 2020; Han et al., 2020). Research using culturomics and microbiome analysis has identified various non-rhizobial endophytes in legume nodules, such as *Pseudomonas, Stenotrophomonas, Bacillus, Enterobacter, Flavobacterium, and Variovorax* (Zhang et al., 2018a; Favero et al., 2020; Han et al., 2020; Tapia-García et al., 2020). Currently, studies on actinorhizal symbiosis have mainly focused on the morphological structure of nodules, isolated cultures of *Frankia*, and the diversity of microbial communities in root nodules. Some studies have suggested that nodules of actinorhizal plants contain more than one species of *Frankia*, and that *Frankia* diversity in nodules may be related to host plant species, the geographical environment, and soil texture, but not to the abundance of *Frankia* in the soil (Tekaya et al., 2018; Balkan et al., 2020). In addition, some non-*Frankia* microbes such as *Streptomyces, Nocardia, and Micromonospora* in nodules, may assist nodulation and promote plant growth (Trujillo et al., 2015; Alekhya and Gopalakrishnan, 2017; Ghodhbane-Gtari et al., 2019; Karthikeyan et al., 2022). However, the interactions among nodule endophytes, nodule surface soil microbes, and geographical environmental characteristics have not been sufficiently investigated.

Sea buckthorn (*Hippophae* L.) is a typical actinorhizal plant that can form nitrogen-fixing root nodule symbiosis with actinobacteria of the genus *Frankia* to provide essential nutrients for its growth and development (Khan et al., 2010). Sea buckthorn is widely distributed in Eurasia, and the global area of sea buckthorn forest is approximately three million hectares, of which 85% are in China. Sea buckthorn is rich in bioactive compounds, including Vitamin C (Hussain et al., 2014; Yu et al., 2022), unsaturated fatty acids (Tkacz et al., 2019; Yu et al., 2022), and flavonoids (Tkacz et al., 2019). Therefore, sea buckthorn has important medicinal value for lowering cholesterol, preventing cardiovascular diseases, repairing scalded skin tissue, and preventing cancer (Olas, 2016; Guo et al., 2017; Masoodi et al., 2020). As a result, research into sea buckthorn has gained increasing attention in recent years. So far, there are two main aspects of research on the diversity of microbial communities in sea buckthorn nodules. The first aspect of research focuses on the isolation of *Frankia* strains from sea buckthorn nodules. *Frankia* strains such as GFN14 (Fernandez et al., 1989), Hr27 (Fernandez et al., 1989), Hr75_2_ (Bautista et al., 2011), and CH37 (Mohr et al., 2020) have been successfully isolated from nodules. Additionally, other endophytes, like *Bacillus*, *Nocardia*, *Micromonospora*, and *Streptomyces*, can also be isolated from sea buckthorn nodules (Luo et al., 2021; Wei et al., 2022). The second aspect of research involves analyzing the microbial community composition of sea buckthorn nodules and rhizosphere using 16S rRNA gene amplicon sequencing. Studies have revealed that sea buckthorn nodules contain a diverse range of microorganisms, but their alpha diversity is significantly lower compared to the rhizosphere. Furthermore, the dominant populations in the rhizosphere and sea buckthorn nodules exhibit variations (Zhang et al., 2018b, 2021). Despite these findings, our knowledge of the composition and function of the sea buckthorn nodule microbial community, the mechanisms of interaction between the nodule microbial community and host are still limited.

Herein, we employed both 16S rRNA gene sequencing and metagenomic sequencing methods to compare and analyze the microbial community composition and differences in nitrogen cycling pathways of sea buckthorn root nodule (RN), nodule surface soil (NS), and bulk soil (BS) grown under three distinct ecological conditions in northern China. Meanwhile we also analyzed the diversity characteristics of different microbial communities and main influencing factors combined with the data for climate and soil environmental factors in the planting area. This study will respectively construct the correlation network of sea buckthorn RN, NS and BS microbial community analyse the stability of microbial community in RN, NS and BS, and explore a dominant strain could exist in RN, serving as the main contributor to nitrogen fixation. The findings of this study will represent the first exploration of the effects of diverse ecological and geographical environments on the microbial communities associated with sea buckthorn RN. This study will contribute to the advancement of research on microbial interactions within actinorhizal plant nodules.

## 2 Materials and methods

### 2.1 Materials and study site description

The Mongolian cultivar of sea buckthorn (*Hippophae rhamnoides* ssp. Mongolica ‘Shenqiuhong’, ‘SQH’) was used as experimental material, which has been widely planted in the ‘Three North Region’ of China in recent years. All analyzed samples were collected from sea buckthorn plantations in three planting areas of Dengkou County (DK) in the Inner Mongolia Autonomous Region, Fuxin Mongolian Autonomous County (FX) in Liaoning Province, and Suiling County (SL) in Heilongjiang Province (**Supplementary Figure S1A**). Dengkou, Inner Mongolia Autonomous Region belongs to typical temperate semi-arid continental monsoon climate characteristics. The mean annual temperature in this region can reach 9.1 °C with a total annual precipitation of only 262.6 mm, and dry and barren sandy loam soil. Fuxin Mongolia Autonomous, Liaoning Province belongs to a temperate continental monsoon climate, with a total annual precipitation of about 555.9 mm, a mean annual temperature of 8.8°C, and sandy loam soil with good air permeability. The third site was Suiling, Heilongjiang Province, which has a typical North temperate continental monsoon climate. The total annual precipitation is 645.5 mm, with a mean annual temperature of 2.3 °C, and nutrient-rich loamy soil (**Supplementary Table S1**).

### 2.2 Sample collection and processing

Three sea buckthorn plants with similar growth conditions were selected from each sea buckthorn plantation in each planting area. To reduce the interaction between plants, keep each plant at least 20 m away from each other. Using the whole plant excavation method to collect samples of sea buckthorn root nodule (RN), nodule surface soil (NS, <1 mm, the distance from the surface of the RN was less than 1 mm), and bulk soil (BS, >1 mm) (**Supplementary Figures S1B, C**). Due to the randomness of the RN position of sea buckthorn roots, 3–5 RNs were collected from each plant, and then the 1-mm-thick soil layer attached to the nodule surface was collected and mixed as the NS samples. The collected fresh RN samples were rinsed three times with sterile distilled water, then vortexed and soaked with 75% (v/v) ethanol solution for two minutes. Next, samples were transferred to sodium hypochlorite solution (2% v/v) vortexed, and soaked twice for five minutes. Finally, RN samples were rinsed five times with sterile distilled water. The sterile distilled water used in the final nodule surface sterilization rinse was then used to spread on yeast-mannitol-agar plates. Subsequently, plates were incubated at 28 °C for 48 h to confirm their sterility and stored as samples for later analysis. Fresh RN, NS and BS samples were frozen with liquid nitrogen and stored at -80 °C for metagenomic analysis. Meanwhile, half of the BS samples were dried for further soil composition analysis.

### 2.3 Analysis of environmental factor characteristics

The climatic factors studied mainly include the mean annual temperature (MAT) and the total annual precipitation (TAP). The data was based on the 10-year average observation data obtained from the meteorological station established in the three sea buckthorn plantations in the three planting areas. Soil physicochemical properties including soil pH, soil organic matter (SOM), total nitrogen (TN), available nitrogen (AN), total phosphorus (TP), available phosphorus (AP), total potassium (TK), available potassium (AK), nitrate nitrogen (NO_3_-N), ammonium nitrogen (NH_4_-N) were measured according to the method detailed in the Handbook of Soil Analysis (Pansu and Gautheyrou, 2007).

### 2.4 DNA extraction, 16S amplicon sequencing, and data processing

Genomic DNA was extracted from surface-sterilized RN, NS, and BS samples using the E.Z.N.A.^®^ Stool 177 DNA Kit (D4015-02, Omega, Inc., USA) according to the manufacturer’s protocol. The concentration and purity of the DNA were quantified by agarose gel electrophoresis and a Nanodrop 2000 spectrometer, respectively. To construct 16S rRNA sequencing libraries, forward primer 338F (5′-ACT CCTACGGGAGGCAGC A-3′) and the reverse primer 806R (5′-GGACTACHVGGGTWTCTAAT-3′) were used for PCR amplification of the bacterial 16S rRNA gene V3-V4 region. A Nanodrop 2000 spectrometer was used to evaluate the quality and concentration of the PCR products. 27 sequencing libraries of 16S rRNA were prepared and then sequenced on the Illumina NovaSeq platform for 250-bp paired-end reads at LC-BIO TECHNOLOGIES CO., LTD. (Hangzhou, China). First, the raw reads sequenced were filtered using Trimmomatic v0.33 software (Bolger et al., 2014). Then cutadapt 1.9.1 software (Martin, 2011) was used to identify and remove primer sequences, and clean reads without primer sequences were obtained. For clean reads obtained previously, dada2 (Callahan et al., 2016) method in QIIME2 software (https://docs.qiime2.org/2021.8/tutoria ls/moving-pictures/) was performed to denoise, paired-end sequence splicing and removing chimeric sequences, obtaining the final valid data (Non-chimeric Reads) amplicon sequence variants (ASVs). Finally, the obtaining ASVs were filtered with a threshold of 0.0001%. The taxonomic identity of ASVs were obtained by aligning them against the SILVA database using QIIME2.

### 2.5 metagenomics sequencing, and data processing

After passing the quality inspection, 27 DNA libraries were sequenced on the Illumina HiSeq 4000 platform for 150-bp paired-end reads at LC-BIO TECHNOLOGIES CO., LTD. (Hangzhou, China). Sequencing reads were adapter-trimmed using the cutadapt v1.9 (Martin, 2011) from the raw sequencing reads. Low-quality reads were trimmed via fqtrim v0.94 (Pertea, 2015), and clean reads were aligned to the host plant genome utilizing bowtie2 v2.2.0 (Langmead and Salzberg, 2012) to eliminate host plant contamination. The quality-filtered reads were *de novo* assembled to construct the metagenome for each sample by IDBA-UD v1.1.1 (Peng et al., 2012). All coding region sequences (CDS) of metagenomic contigs were predicted using MetaGeneMark v3.26 (Zhu et al., 2010), and CDS of all samples were clustered using CD-HIT v4.6.1 (Fu et al., 2012) to obtain unigenes. The unigene abundance for a certain sample was estimated by transcripts per kilobase million (TPM) based on the number of aligned reads determined in bowtie2 v2.2.0. The functional annotation and taxonomic identity of unigenes were obtained by aligning them against the NCBI NR database using DIAMOND v0.9.20 (Buchfink et al., 2015).

### 2.6 Statistical analyses

One-way analysis of variance (ANOVA) was performed to analyze the significance of the difference in microbial community diversity, microbial abundance, nitrogen cycle abundance, and environmental factors. The Origin 2019 pro software was used for visual presentation. PCoA analysis based on Bray-Curtis distances and plotting were performed using the vegan package, and the PERMANOVA was applied to determine whether significant differences existed in microbial community composition. A *p* value less than 0.05 was considered statistically significant. To explore and analyze the potential interactions between different microorganisms, we selected the top 30 dominant microorganisms at the genus level to construct interaction networks. Network analysis was performed using the igraph1.3.5 package in R. Spearman correlations were calculated using the stats package in R and only robust significant interactions (r > 0.6 or r < -0.6; *p* < 0.05) were retained for network analysis. The linear discriminant analysis (LDA) effect size (LEfSe) was generated using LEfSe software (Segata et al., 2011), with a logLDA score >4.0 and *p* < 0.05, to identify significant differences in microbial taxa among RN, NS, and BS. Partial Mantel tests were performed using the linkET package in R to detect the correlations between environmental factors and microbial community structures. We used the stats package in R to calculate Spearman’s correlation between environmental factors and high abundance microbes and the ggplot2 package in R was used to generate the correlation heatmaps (Team R.C, 2020).

## 3 Results

It is largely known that root nodule was the Actinorhizal plants form a special niche for nitrogen fixation. In the sea buckthorn ecosystem, knowing the diversity and function of the nodule-associated fine-scale microbial community are the key to clarify the assembly mechanism of microbial community and their ecological importance. In this study, 16S rRNA and metagenomic sequencing were used to reveal the differences in microbial diversity and nitrogen cycling function in the three nodular compartments, and analyzed the effects of environmental factors on different sample types combined with environmental data.

### 3.1 Microbial community diversity from soil to root nodule decreased significantly

**Figure 1:**
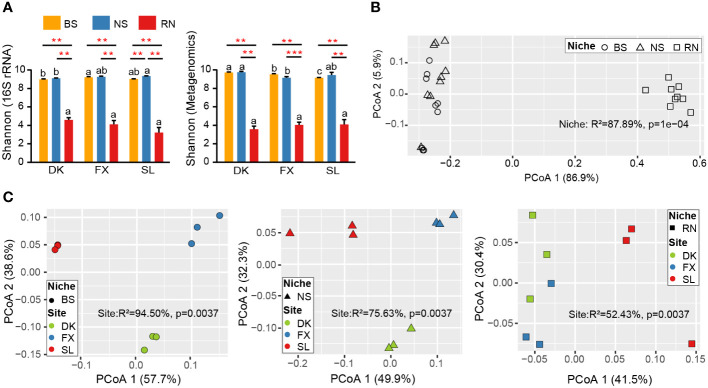
Microbial community diversity of root nodule (RN), nodule surface soil (NS) and bulk soil (BS) at three planting areas. **(A)** Histograms showing the differences of alpha diversity index for microbial communities from the BS, NS, and RN in three sites based on 16S rRNA data and metagenomic data, respectively. Different lowercase letters on the top of histograms indicate significant differences among samples at different sites. The short solid line represents the ANOVA analysis among different samples in the same site. The asterisk above the short solid line represents a significant difference among different samples, **p < 0.01, and ***p < 0.001. **(B)** Principal coordinate analysis (PCoA) of metagenomic data based on Bray-Curtis distance showing the microbial community composition of BS, NS, and RN in three sites. **(C)** PCoA of metagenomic data showing the impact of site on the composition of BS, NS, and RN microbial communities, respectively. PERMANOVA was used to analyze the impact of different grouping factors on sample differences.

We performed 16S amplicon sequencing and metagenomic sequencing on 27 samples collected. 16S amplicon sequencing data through quality filtering and DADA2 de-noising, a total of 1596598 final valid data was obtained. After filtering, we ultimately identified 10545 ASVs for 16S rRNA (**Supplementary Table S2**). After removing sea buckthorn genome sequences (Yu et al., 2022), more than 3.03 billion shotgun genome sequences were generated, with an average of about 114 million per sample (**Supplementary Table S3**). Subsequently, the metagenomes were aggregated into about two million non-redundant genes (unigenes). To determine the distribution characteristics of microbial diversity in three different samples of RN, NS, and BS, we calculated the Shannon index based on 16S amplicon and metagenomic data respectively. Similar results were observed in PCoA analyses of 16S amplicon sequencing data and metagenomic sequencing data. The result revealed that the microbial communities in BS and NS exhibited significantly higher diversity compared to RN samples, whereas no significant difference was observed between NS and BS (**Figure 1A**), and these findings remained consistent across the three sampling sites in diverse eco-geographical environments. By comparing the differences in alpha diversity across the three sampling sites (**Figure 1A**), we observed a significant variation in the diversity indices of BS between different sites. Furthermore, the Shannon index of NS was found to be significantly different between the DK and SL sites (16S rRNA), or between the DK and FX sites (Metagenomics). However, the alpha diversity indices of RN did not exhibit significant differences among the three sampling sites with different eco-geographical environments, suggesting no association between the microbial community diversity and changes in eco-geographical environments within the sea buckthorn RN. Based on the Bray Curtis distance, the results of PCoA and PERMANOVA analysis further showed that the compartment niche had a significant effect on microbial community composition (R^2^ = 87.89%, *p*=1e-04; **Figure 1B**; R^2^ = 39.72%, *p*=1e-04; **Supplementary Figure S2A**). Samples from the same compartment niches were significantly different among the three sites (**Figure 1C**; **Supplementary Figures S2B–D**). Furthermore, the PCoA analysis of metagenomic data showed (**Figure 1C**), the variance contribution rate of the site to microbial community composition in compartment niches gradually decreased (BS>NS>RN). The variance contribution rate of compartment niches to microbial community composition was 87.89%, which was higher than that of site to NS (75.63%) and RN (52.43%), but lower than that of site to BS (94.50%). This indicated that the microbial community composition of NS and RN was more influenced by niche compartment, while BS was more influenced by site.

### 3.2 Root nodules have a significant selective filtration effect on soil microbial community

**Figure 2:**
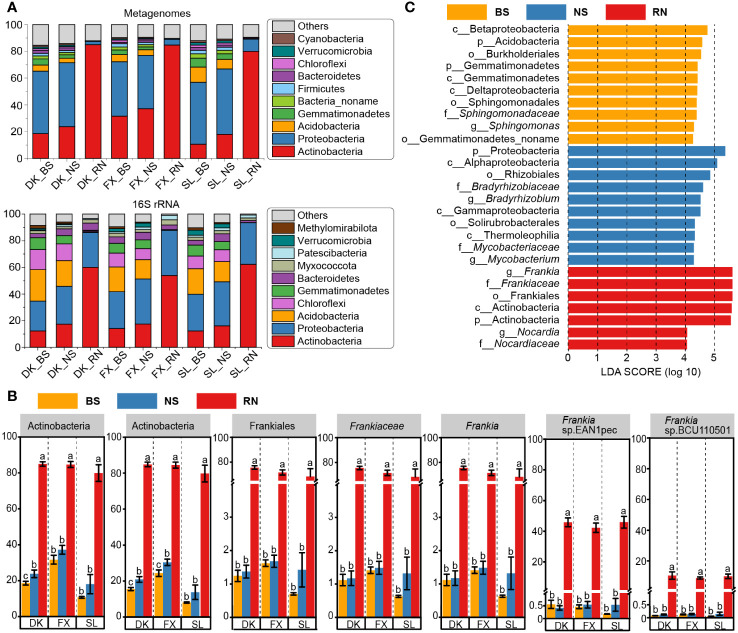
Comparison of differences in the composition of microbial communities in RN, NS and BS in three different planting areas. **(A)** Microbial community composition (phyla level) in bulk soil (BS), nodule surface soil (NS), and root nodule (RN) from 16S rRNA amplicon sequencing data and metagenomics data. **(B)** Linear discriminant analysis effect size (LEfSe) tool was used to identify biomarker taxa (taxa with LDA score > 4) associated with RN, NS, and BS respectively. **(C)** Differences in abundance of major Actinobacteria microorganisms in RN, NS and BS. At the level of 0.05, the variance analysis showed that the differences among the three groups were statistically significant. Different lowercase letters indicate significant differences among different samples.

Differences in microbial community composition showed that the community composition of 16s amplicon and metagenomic sequences was similar at the phylum level. The microbial community in RN consisted mainly of Actinobacteria, with lesser amounts of Proteobacteria (**Figure 2A**). In comparison, Proteobacteria was the main microbial phyla in NS and BS, closely followed by Actinobacteria and Acidobacteria. Moreover, Gemmatimonadetes, Firmicutes, Bacteroidetes, and Chloroflexi were also present in NS and BS microbial communities, but their abundance in RN was extremely low. Furthermore, we observed a higher proportion of Actinobacteria in NS compared to BS, implying that NS, serving as the transitional zone between RN and BS, also fostered Actinobacteria enrichment. Statistical analysis of the proportions of Actinobacteria on different taxonomic levels in different types of samples (**Figure 2B**; **Supplementary Table S6**) also found that the enrichment of highly diverse Actinobacteria in RN was mainly dominated by Actinobacteria (79.76%–84.85%) - *Frankia*les (68.38%–75.61%) - *Frankiaceae* (68.31%–75.53%) - *Frankia* (68.31%–75.53%), while the proportion of other Actinobacteria in RN was very low. LEfSe results showed that the genus *Frankia* was the most significant biomarker taxa in RN (**Figure 2C**), thus suggesting that the distinction between microbial communities of RN and the soil (NS and BS) was mainly determined by *Frankia*. Statistical results showed that *Frankia* sp. EAN1pec was dominant in RN among the top four species in *Frankia* genus, accounting for 42.27%-45.92% of the total abundance and 59.09%-67.22% of the abundance of the *Frankia* genus. The abundance of *Frankia* sp. EAN1pec in RN was significantly higher than BS and NS (*p*<0.05) (**Figure 2B**; **Supplementary Table S4**). However, *Frankia* sp. BCU110501, the second most abundant, accounted for only about 13.61% of the RN, and the other *Frankia* species were even less abundant. This indicates that *Frankia* sp. EAN1pec is more important in sea buckthorn RN compared to other *Frankia* species of the same genus and is a specific actinomycete associated with the formation of sea buckthorn RN.

**Figure 3:**
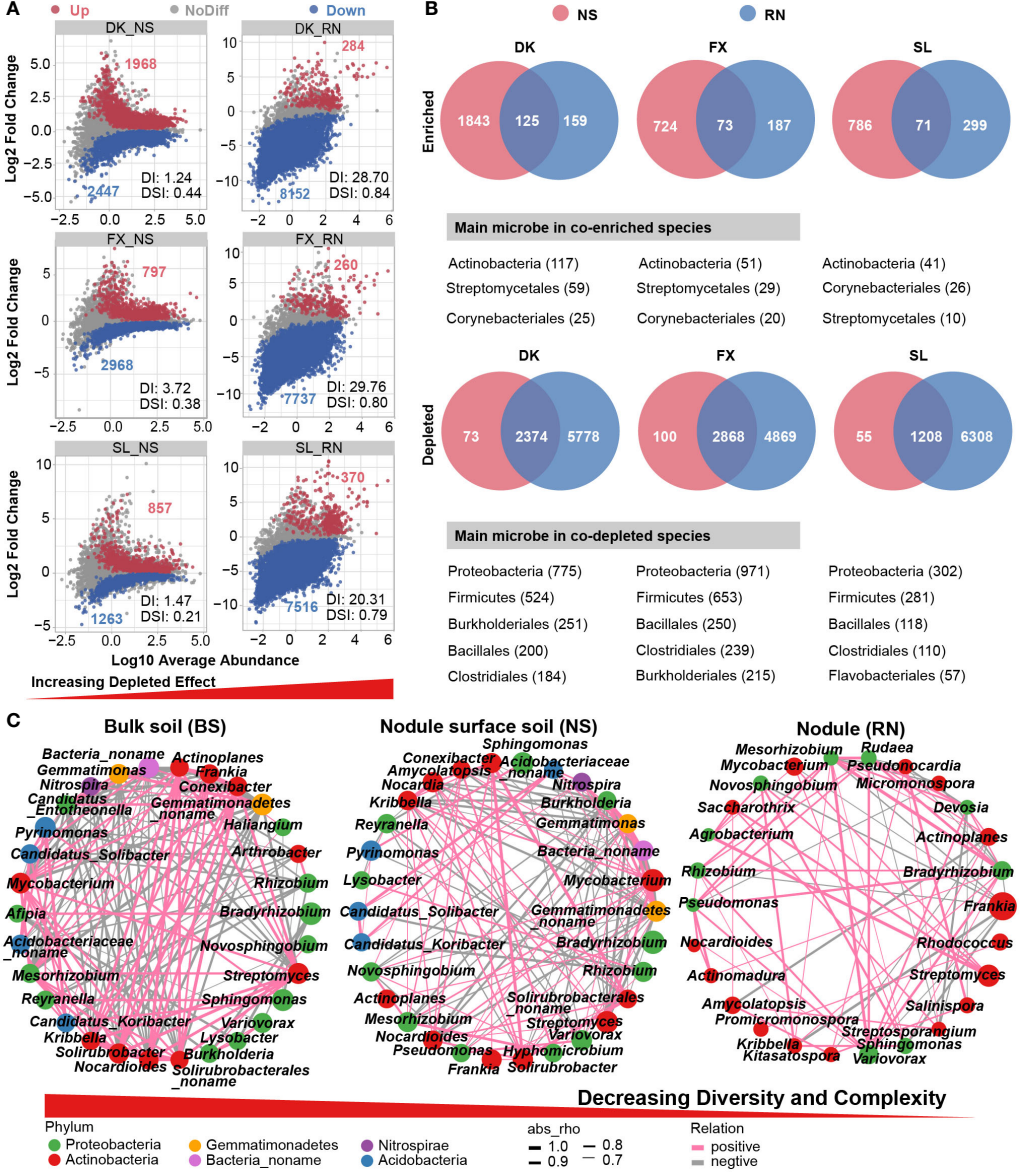
Microbial interaction networks and enriched/depleted microbial species in bulk soil (BS), nodule surface soil (NS), and nodule (RN). **(A)** Enriched and depleted species in RN and NS compared to the BS. Each point represents an individual species, and the position along the y-axis represents the abundance fold change. **(B)** Venn diagrams showing the number of shared and exclusive microbial species in different compartment within the significant enriched species and depleted species. These shared differentially expressed species are shown below the Venn diagram (only phyla and orders with high abundance are shown). **(C)** Interaction network of dominant microbiota at the genus level (top 30) in the BS, NS and RN. The size of the nodes shows the abundance of the genus, and the different colors indicate the corresponding taxonomic assignment at the phylum level. The edge color represents positive (pink) and negative (gray) correlations. The edge thickness indicates the correlation values; only significant interactions are shown (|r|> 0.6; p < 0.05).

We performed differential abundance analyses to identify the species that contributed to the divergence in microbial community composition among RN, NS, and BS. Compared with BS, we detected a significant increase in the number of differential species from NS to RN in three sites, such as 4415-8436 in DK_NS-RN, 3765- 7997 in FX_NS-RN, and 4415-8436 in SL_NS-RN (**Figure 3A**), accompanied by a significant decrease in the number of enriched species in RN. The results of the differential abundance analysis revealed a significant enrichment and overlap of microbial communities, primarily belonging to the orders Streptomycetales and Corynebacteriales in Actinobacteria in both RN and NS (**Figure 3B**). Conversely, the microbial community, mainly from phyla Proteobacteria and Firmicutes, exhibited significant depletion in both RN and NS, whereas BS showed the opposite pattern. We further evaluated the selective filtration process of microbial species from BS to NS to RN by utilizing the Depleted index (DI) and the Dissimilarity index (DSI) previously defined in studies (Xiong et al., 2021). The two indices gradually increased from NS to RN, indicating that the depletion effect gradually increased from BS to NS to RN, while the microbial community difference in BS also gradually increased. It was obvious that RN played a significant role in microbial selection and filtration.

### 3.3 The correlation and complexity of the microbial association network from soil to nodule decreased significantly

To understand the selection process of microbial community from soil to nodule, we analyzed the symbiotic network involving the top 30 genera. The analysis revealed 153 correlations in BS, 122 correlations in NS, and 75 correlations in RN (**Figure 3C**; **Supplementary Tables S5**–**S7**). As expected, the interaction networks of NS and RN gradually simplified when compared to the BS network. The decrease in correlations between microbes was accompanied by a gradual reduction in the complexity of the microbial network from BS to RN. The symbiotic networks in BS and NS both contained six different phyla, while RN contained only two phyla, with a higher proportion of nodes belonging to Actinobacteria (16 genera). The dominant genus *Frankia* was negatively correlated with eight genera, especially *Sphingomonas* (-0.87) and *Kribbella* (-0.92), indicating that although these microbial taxa were associated with the plant symbiont, they were mutually exclusive with *Frankia*. This may be attributed to the capacity of sea buckthorn to form a symbiotic relationship with *Frankia*, resulting in a high abundance of *Frankia* in the nodules and impeding the colonization of root nodules by other microbes. This may also be due to that they are related to other tissues in the plant or have overlapping functions, causing them to be negatively correlated with *Frankia* in the nodule.

### 3.4 The abundance of *Frankia* sp. EAN1pec in root nodules is not related to changes in the eco-geographical environment

**Figure 4:**
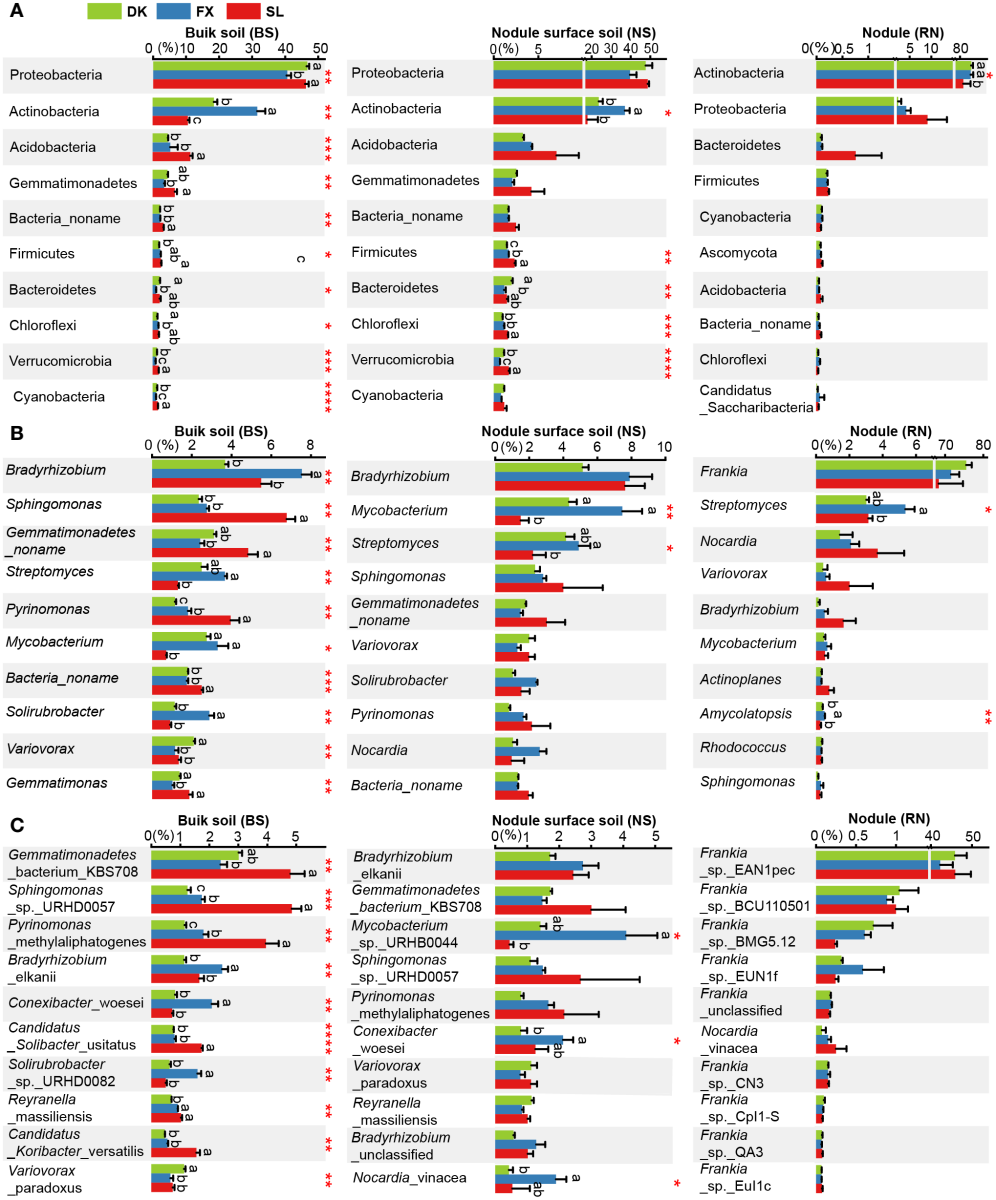
Comparison of microbial community composition in three different planting areas. Comparison of relative abundances of **(A)** major phyla (top 10 phyla), **(B)** major genera (top 10 genera) and **(C)** major species (top 10 species). Different lowercase letters on the top of bars indicate significant differences among different sites. ANOVA was used to evaluate the significance of differences between the indicated groups (* indicates p < 0.05; ** indicates p < 0.01; *** indicates p < 0.001;**** indicates p < 0.0001).

**Figure 5:**
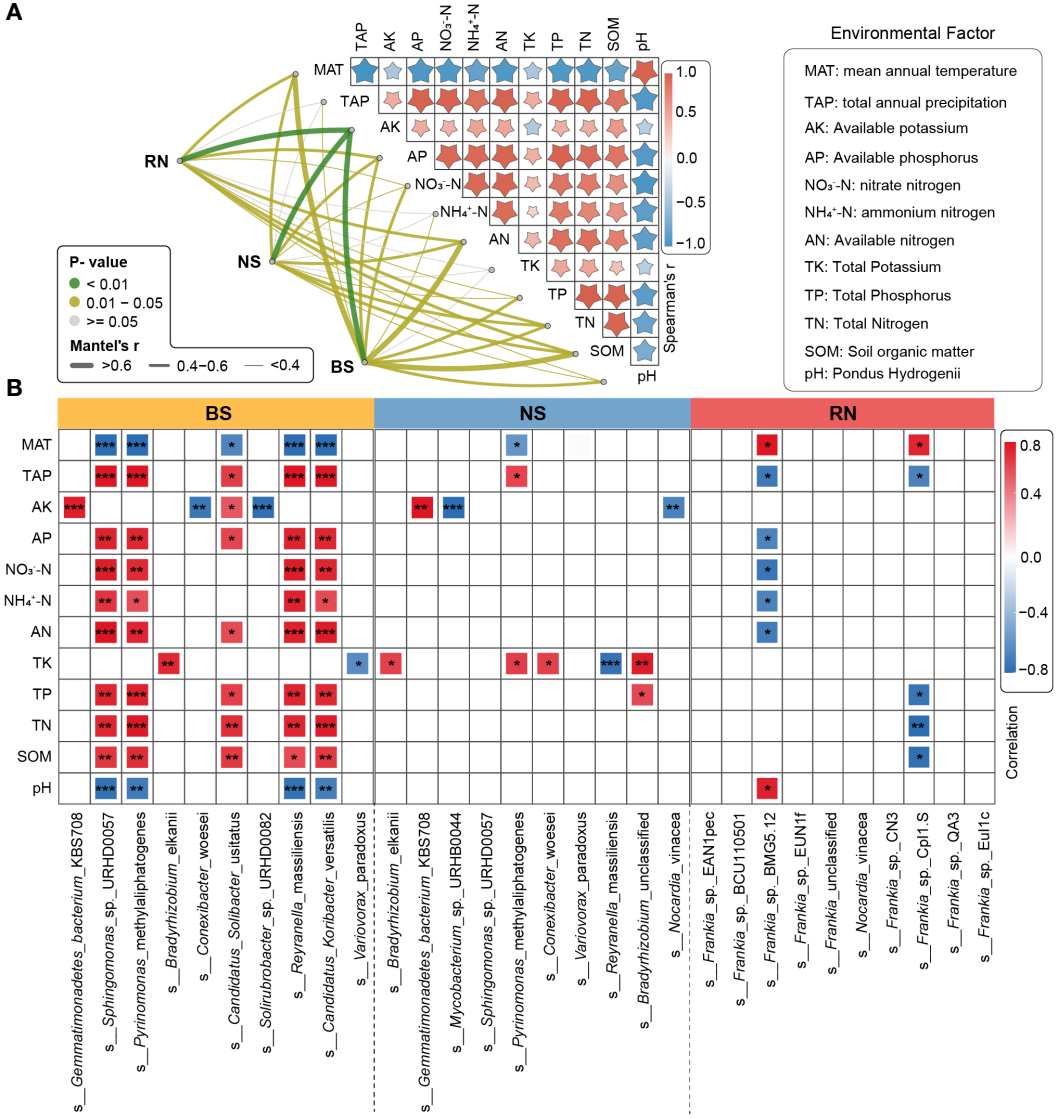
Environmental factors affecting microbial communities in different compartment niches. **(A)** Microbial community compositions of different compartment niches were correlated with environmental factors based on the partial Mantel tests. Pairwise comparisons of environmental factors are displayed with a color gradient to denote Spearman’s correlation coefficients. The line width corresponds to the Mantel r-value and the line color indicates the statistical significance based on 999 permutations. **(B)** Spearman correlation analysis between top10 species and environmental factor. The significance of correlation analyses is marked with asterisks (*) at different significance levels (* for p < 0.05, ** for p < 0.01 and *** for p < 0.001). SOM, Soil organic matter; pH, Pondus Hydrogenii; TN, Total Nitrogen; TP, Total Phosphorus; TK, Total Potassium; NO_3_^–^N, nitrate nitrogen; NH_4_^+^-N, ammonium nitrogen; AN, Available nitrogen; AP, Available phosphorus; AK, Available potassium.

In the three different planting areas, the relative abundance of the top 10 microbial phyla in the BS microbial community exhibited significant variation. The relative abundance of Proteobacteria in BS of FX was significantly lower compared to DK and SL, while relative abundance of Actinobacteria in BS of FX was significantly higher compared to DK or SL. Only five phyla in the NS microbial community exhibited significant differences among different sites, including Firmicutes, Bacteroidetes, Chloroflexi, Verrucomicrobia and Actinobacteria. No significant differences were identified in the main phyla Proteobacteria in NS. In the RN microbial community, only the relative abundance of Actinobacteria exhibited significant differences between the different sites (**Figure 4A**). At the genus level, the relative abundance of the top 10 microbial genera in the BS microbial community showed significant differences (**Figure 4B**). Only two genera, *Mycobacterium* and *Streptomyces*, were significantly different in the NS microbial community. Moreover, only two genera, *Streptomyces* and *Amycolatopsis*, were significantly different in the RN microbial community. At the species level, the relative abundance of the top 10 microbial species in the BS microbial community were all significantly different. Only three species in the NS microbial community exhibited significant differences. This suggested that even the composition of the NS was largely influenced by the root nodules, regardless of the eco-geographical regions. Remarkably, the abundance of *Frankia* sp. EAN1pec, the dominant microbial species in RN microbial community, did not show significant differences among the three different planting areas (**Figure 4C**). This suggested that the relative abundance of *Frankia* sp. EAN1pec in sea buckthorn RN was not significantly affected by the different eco-geographical environments. In addition, we also analyzed the differences in the abundance of all 19 *Frankia* species contained in RN at different sites (**Supplementary Table S8**). These 19 *Frankia* species were distributed in different *Frankia* clusters (Hong et al., 2012; Nouioui et al., 2019; Gtari, 2022). We observed that most of the *Frankia* species in the RN microbial community did not differ significantly between the different sites, but their relative abundance was very low. For example, *Frankia* sp. BCU110501, whose native host plant was *Discaria trinervis* (*Rhalmnaceae*), belongs to group III with *Frankia* sp. EAN1pec and had a relatively large genome. Its abundance had no significantly between different sites, but its relative abundance was much lower than *Frankia* sp. EAN1pec (Wall et al., 2013). These results suggested that these *Frankia* species were not susceptible to environmental influences in the stable environment of the root nodules. To investigate the factors contributing to disparities in microbial community composition, we further examined the effects of environmental factors on microbial communities. Our findings showed significant differences in each environmental factor across the three sea buckthorn planting areas (**Supplementary Table S9**). The Mantel test analysis revealed the strongest correlation between AK and BS, NS and RN microbial communities (r > 0.6, p < 0.01; **Figure 5A**). Eight environmental factors (MAT, AN, SOM, TN, AP, TP, NO_3_^–^N and pH) were significantly correlated with BS microbial composition (r>0.4, *p*<0.05, **Figure 5A**; **Supplementary Table S10**), with MAT, AN, and SOM exhibiting a higher correlation with BS microbial composition (r>0.6, *p*<0.05). Five environmental factors (AN, MAT, SOM, AP and TN) showed significant correlations with microbial composition in both NS and RN (r > 0.4, p < 0.05). These results indicated a complex relationship between the microbial community composition of RN, NS and BS, and environmental factors. We further analyzed the relationship between environmental factors and high-abundance strains. There were significant correlations between the top 10 microbial strains in the BS microbial community and various environmental factors. In the NS microbial community, eight species showed significant correlations with environmental factors, but with lower complexity than in the BS microbial community. Moreover, only two species in the RN microbial community demonstrated significant correlations with environmental factors (**Figure 5B**). This suggested that the BS microbial community was more vulnerable to numerous eco-environmental factors than those in RN and NS. Notably, *Frankia* sp. EAN1pec, which was the most abundant strain in the RN microbial community, did not exhibit any significant correlation with environmental factors. Similarly, *Frankia* sp. EAN1pec in NS microbial communities also had no significant correlation with environmental factors. However, in the BS microbial community, *Frankia* sp. EAN1pec was significantly correlated with several environmental factors (**Supplementary Table S11**). These results suggested that *Frankia* sp. EAN1pec was more stable in RN and NS microbial communities than in BS. In addition, *Frankia* sp. BCU110501, which ranked second in abundance, and most of the low-abundance *Frankia* were not affected by environmental factors in RN, which also indicated that *Frankia* bacteria were not easily affected by environmental factors in root nodules.

### 3.5 Significant differences in microbial nitrogen cycling pathways among bulk soil, nodule surface soil, and root nodule

**Figure 6:**
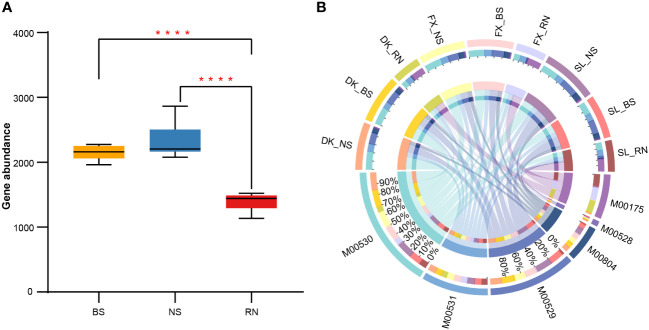
Comparison of nitrogen cycle abundance. **(A)** Boxplots showing differences in the overall nitrogen cycle abundance in bulk soil (BS), nodule surface soil (NS), and nodule (RN). **(B)** The distribution of different nitrogen cycle pathways in BS, NS, and RN. Data were visualized using Circos. The length of the bars of compartments on the inner-ring represented the percentage of each nitrogen cycle. M00175: nitrogen fixation; M00528: nitrification; M00529: denitrification; M00530: dissimilatory nitrate reduction; M00531: assimilatory nitrate reduction; M00804: complete nitrification. ANOVA was used to evaluate the significance of differences between the indicated groups (**** indicates p < 0.0001).

Nitrogen is an essential component for plant growth, which can be fixed by nitrogen-fixing root nodules and subsequently provided to plants. Therefore, by comparing changes in microbial nitrogen cycle pathways in RN, NS, and BS, we can better understand the nitrogen fixation mechanism of plant-microbial symbionts. To gain a better understanding of the nitrogen fixation mechanism in plant-microbial symbionts, we compared the changes in microbial nitrogen cycle pathways in RN, NS, and BS. Our metagenomic contigs were annotated using the Kyoto Encyclopedia of Genes and Genomes (KEGG) databases. We found significant differences in the function of RN, NS, and BS microbial communities. Comparing the total nitrogen cycle pathways in RN, NS, and BS microbial communities, we discovered that the abundances of total nitrogen cycle pathways detected in RN was significantly lower than in the NS and BS, but there were no significant differences in the abundance of total nitrogen cycle pathways in the NS and BS (**Figure 6A**). Further analysis of six different nitrogen cycling pathways, including dissimilatory nitrate reduction, assimilatory nitrate reduction, nitrogen fixation, denitrification, complete nitrification, and nitrification, showed significant differences in the abundance of different nitrogen cycling pathways between different samples (**Figure 6B**; **Supplementary Table S12**). In general, dissimilatory nitrate reduction (M00530, 32.74%) was the most common microbial nitrogen cycle pathway, followed by denitrification (M00529, 21.60%), assimilatory nitrate reduction (M00531, 19.00%), and nitrogen fixation (M00175, 16.64%). Both complete nitrification (M00804, 8.86%) and nitrification (M00528, 1.16%) were detected in low abundance. In addition, the distribution of six different N cycle pathways was also significantly different among RN, NS, and BS samples (**Figure 6B**). The totality of nitrate reduction account for 85.03% and 84.56% of gene abundance of microbial nitrogen cycle-related pathways in BS and NS, respectively. However, the abundance of nitrification was low in all samples. The abundance of nitrogen fixation genes in BS and NS was very low (0.79%–2.16%), while the abundance of nitrogen fixation genes in RN reached 47.68% on average, indicating that RN was the main nitrogen fixation organ in sea buckthorn. In addition, we found that assimilatory nitrate reduction and dissimilatory nitrate reduction accounted for 22.86% and 23.57% of nitrogen cycling abundance in RN, respectively (**Figure 6B**; **Supplementary Table S12**).

The analysis results of major contributing strains of six nitrogen cycling pathways in RN, NS, and BS microbial communities are presented in **Supplementary Table S11**. Proteobacteria and Actinobacteria were the main microbial groups in each nitrogen cycling pathway in BS and NS microbial communities, but no major contributing species were found. The top 10 strains contributing to nitrogen fixation differed greatly in RN, NS, and BS. Among them, *Frankia* sp. EAN1pec was the main contributor in RN, accounting for 81.9%–96.9% (**Supplementary Table S13**), while the abundance of nitrogen-fixing genes of other strains in RN, NS and BS was very low. Furthermore, we found that *Frankia* sp. EAN1pec was most dominant in both the dissimilatory nitrate reduction and the assimilatory nitrate reduction pathway in RN, accounting for 60.5%–79.1% and 50.0%–57.3%, respectively (**Supplementary Table S13**). However, *Frankia* sp. BCU110501 and *Frankia* sp. BMG5.12 only accounted for about 13.6%-33.1% and 6.1%-17.1% respectively in assimilatory nitrate reduction pathway in RN. *Nocardia vinacea* only accounted for 2.0% - 5.5% in dissimilatory nitrate reduction pathway in RN. These results indicated that in RN, *Frankia* sp. EAN1pec can not only provide nitrogen to plants by nitrogen fixation, but also might provide nitrogen for plants by nitrate reduction.

## 4 Discussion

The RN is a special nitrogen-fixing organ formed by a symbiotic relationship between plant roots and *Frankia* Actinomyces. Analyzing the diversity characteristics of sea buckthorn RN microbial community and its relationship with changes in the ecological environment has important scientific value for elucidating the formation of RN and the mechanism of nitrogen fixation in actinorhizal plants.

### 4.1 The host plant (sea buckthorn) determines the unique microbial composition of root nodules

Previous studies have demonstrated that both the rhizosphere and endophytic regions of plants have a core microbiome. The composition of these microbial communities depends on host genotype and ecological niches (Cregger et al., 2018; Xiong et al., 2021). We found that environmental factors had a significant influence on the BS microbial community, but had an insignificant or weak effect on RN and NS microbial communities (**Figure 5**). Notably, *Frankia* sp. EAN1pec, the most dominant species in RN microbial community, was not associated with changes in ecological environmental factors (**Figure 5B**). These results suggested that RN microbial communities of sea buckthorn can still maintain relatively stable under different ecological environmental conditions, which is mainly determined by the host plant, and is not affected by changes eco-geographical environment. Our findings differ from the conclusions drawn in previous studies. Research on rhizobia symbioses showed that the symbiotic nitrogen-fixing bacteria (mainly *Bradyrhizobium* and *Sinorhizobium*) in legume nodules are significantly shaped by a range of environmental factors, including soil pH, water status, available iron, and regional climate conditions, exhibiting a strong biogeographical pattern (Han et al., 2009; Ferguson and Gresshoff, 2015; Zhang et al., 2017; Sharaf et al., 2019). Studies of actinorhizal symbioses showed that the symbiotic nitrogen-fixing actinomycetes (mainly *Frankia*) in root nodules are also affected by the environment. It was found that the relative abundance of *Frankia* in Casuarina nodules was strongly influenced by environmental gradient (Ghodhbane-Gtari et al., 2021). In the humid climate zone, *Frankia* was the most dominant genus occupying up to 80% of the nodule of *Casuarina glauca*, while in semiarid and arid environments, the abundance of *Frankia* in the nodule was drastically reduced. A study of *Frankia* diversity within the alder root nodules on Mount St. Helens revealed that small-scale geographic heterogeneity can affect the host-specificity of *Frankia* communities in red alder (*Alnus rubra*) and Sitka alder (*Alnus viridis*) (Wolfe et al., 2022).Soil type was also shown to have a significant impact on *Frankia* diversity within host nodules (Welsh et al., 2009; Pokharel et al., 2011).

However, more research supports host plants selectively shaping root nodule microbial communities. It has been confirmed that commensal bacteria in the *Lotus* showed obvious host preference (Wippel et al., 2021). There were significant differences in the *Frankia* community composition and structure in the root nodules between the different alders growing on the same soil, indicating that the host plant genotype had a significant effect on the composition and abundance of *Frankia* communities (Vemulapally et al., 2022; Yuan et al., 2023). The study of global biogeography of alder root nodule *Frankia* actinobacteria have also shown that the host is the main determinant affecting the composition of *Frankia* communities in nodule (Põlme et al., 2014). The symbiotic relationship between sea buckthorn and *Frankia* sp. EAN1pec was not easily affected by the changes in the eco-geographical environment, suggesting a more specific and stable symbiosis. This is consistent with previous findings that have highlighted the extensive symbiotic relations and wide environmental adaptability of *Frankia* sp. EAN1pec within the Elaeagnus (Normand et al., 2007). This may result from interactions and coevolution between sea buckthorn and *Frankia* sp. EAN1pec in nature. In addition, other studies have also demonstrated that the genome size of *Frankia* strains, such as CcI3 (5.43 Mbp), ACN14a (7.50 Mbp), and EAN1pec (9.04 Mbp), is associated with both the host-plant range and biogeographical adaptation, especially for *Frankia* sp. EAN1pec, which has a wider range of adaptation to different environments and host due to its larger genome (Normand et al., 2007; Kucho et al., 2014).

### 4.2 Formation of sea buckthorn root nodule is the result of co-evolution of sea buckthorn and soil *Frankia* sp. EAN1pec

In this study, we discovered that the microbial diversity of sea buckthorn RN was significantly lower than that of NS and BS (**Figure 1**). This trend was consistent across the three different growing areas of sea buckthorn, which aligns with findings from previous studies on soybean and alfalfa nodules (Han et al., 2020). Furthermore, we found that different eco-geographical conditions had a significant impact on the microbial community diversity of NS and BS, but had no significant impact on the RN microbial community diversity (**Figure 1**). These results suggested that, despite the large geographical scope of sea buckthorn cultivation, the microbial community diversity of RN remained relatively steady, indicating an intrinsic stable mechanism for the development of sea buckthorn nodules. Further investigation revealed that the diversity indices (DI and DSI) gradually increased from BS to NS to RN, while the complexity of the microbial network decreased (**Figure 3**). This suggests that during the formation of sea buckthorn nodules, sea buckthorn roots have a selective filtration effect on soil microorganisms. Similar selective filtration effects have been reported in the root endosphere and rhizosphere of other plant species, including *Populus* (Beckers et al., 2017), maize, wheat, and barley (Xiong et al., 2021), as well as in the nodule microbiome of soybean and alfalfa (Xiao et al., 2017). Previous studies have confirmed that the selection of rhizosphere and plant endosphere microbial communities is primarily influenced by plant genotype and root exudates, with colonization of these microbial communities may be associated with specific functions (Brown et al., 2020; Wang et al., 2021). Plants have been shown to attract microbial communities involved in the carbon and nitrogen cycle to the rhizosphere or nodule in earlier studies (Pathan et al., 2018). Furthermore, the physical and chemical qualities of soil, as well as changes in the rhizosphere environment, have all been linked to the selective filtering action of plants on microbes (Sharaf et al., 2019; Han et al., 2020).

In this study, the microbial community of sea buckthorn RN showed a unique composition pattern. This was due to the strong selective filtering effect of sea buckthorn roots on soil microorganisms, resulting in *Frankia* being the most dominant genus in the RN (**Figure 2A**). The *Frankia* communities in sea buckthorn RN were distributed in several *Frankia* clusters, including *Frankia* sp. EAN1pec, BCU110501, BMG5.12, EUN1f, etc. At present, the presence of multiple *Frankia* strains in actinorhizal plants nodules has been demonstrated. Studies have shown that there were different *Frankia* strains in the nodules of *Alnus*, *Myricaceae*, and *Coriaria*, and their abundance was also different (Clawson and Benson, 1999; Swanson et al., 2022; Vemulapally et al., 2022). For example, we found that *Frankia* from clusters I, II, and IV were simultaneously found in *Myrica pensylvanica* nodules, suggesting that different *Frankia* clusters could exist in the same host.

Among these *Frankia*, *Frankia* sp. EAN1pec was the most dominant actinomycetes in the sea buckthorn RN, and its abundance was not related to changes in eco-geographical environmental factors. This suggested that *Frankia* sp. EAN1pec was the specific actinomycetes forming sea buckthorn RN (**Figures 2**, **4C**; **Supplementary Table S4**). Despite previous evidence of genus *Frankia* dominance in the RN microbial community (Wei et al., 2022), it was surprising that a single *Frankia* sp. EAN1pec dominated in the RN of sea buckthorn, which was not predicted. *Frankia* sp. BCU110501 occupied the second highest abundance in the RN and belong to the cluster III with *Frankia* sp. EAN1pec, which was much less abundant than *Frankia* sp. EAN1pec. The relative abundance of the other *Frankia* strains in RN was even lower. This showed a more restrictive symbiont specificity between sea buckthorn and *Frankia* sp. EAN1pec (Clawson and Benson, 1999). The most dominant position and stability of *Frankia* sp. EAN1pec in sea buckthorn RN may result from the long-term co-evolution of *Frankia* sp. EAN1pec with sea buckthorn, which is unrelated to *Frankia* sp. EAN1pec abundance in soil (Tekaya et al., 2018). The coexistence of the dominant *Frankia* strain with multiple low-abundance *Frankia* strains may be due to the fact that the multiple functions of genetically diverse *Frankia* can complement each other to improve the overall fitness of host plants in complex ecosystems (Kagiya and Utsumi, 2020). In addition to *Frankia*, we discovered non-*Frankia* microorganisms in sea buckthorn RN (**Figure 4**). Some non-*Frankia* microorganisms, such as *Streptomyces* (Alekhya and Gopalakrishnan, 2017), *Nocardia* (Ghodhbane-Gtari et al., 2019), *Variovorax* (Jiang et al., 2015), and *Mycobacterium* (Wang et al., 2018), have been reported for their ability to assist nodulation and promote plant growth. The presence of non-*Frankia* microorganisms in sea buckthorn RN suggests that they may also play a crucial role in nodule growth and nitrogen fixation processes. However, the specific mechanisms involved remain unclear.

### 4.3 The nitrogen fixation pathway is the main pathway for nitrogen cycling in sea buckthorn RN

Nitrogen availability is an important limiting factor that impacts plant growth and development (Lam et al., 1996). Nitrogen utilization by plants is mainly achieved through microbially mediated processes; therefore, microbes are the primary drivers of nitrogen cycling. Although there have been numerous studies on the involvement of microbes in the nitrogen cycle (Kuypers et al., 2018; Li et al., 2020), there is limited research on the nitrogen cycling specifically associated with the nodule microbes of actinorhizal plants. In this study, we employed metagenomic sequencing to show that the total abundance of the nitrogen cycle in BS and NS microbial communities was significantly higher than in RN (**Figure 6A**). This finding may be closely related to the presence of a more complex microbial community and a wider variety of nitrogen cycling pathways in BS and NS. In contrast, the microbial community structure and nitrogen cycle pathway in RN were relatively simple, with the overall nitrogen cycle abundance significantly lower than in the soil samples (NS and BS). Further, the abundance and composition of the nitrogen cycle in RN microbial community differed substantially from BS and NS. The relative frequency and composition of nitrogen cycle pathways in BS and NS microbial communities appeared to be quite similar across three different sea buckthorn growing areas. Among them, dissimilatory nitrate reduction, denitrification, and assimilatory nitrate reduction were dominant in BS and NS (**Figure 6B**; **Supplementary Table S12**). These results were aligned with previous observations that these pathways are widely distributed in soil samples (Li et al., 2020; Pan et al., 2020). In addition, the contributing microbial groups of each nitrogen cycle pathway in BS and NS were relatively consistent, with Proteobacteria and Actinobacteria being the main microbial groups (**Supplementary Table S13**), although no clear and most dominant species were found. Since the rich nitrate reduction pathways in the BS and NS microbial communities allow for the maintenance of high amino acid biosynthesis, facilitating nitrogen bio-binding and minimizing nitrogen leaching and denitrification losses (Nojiri et al., 2020). While the BS and NS microbial communities also possess nitrogen fixation and nitrification pathways, their abundance were relatively few, which is consistent with previous research (Xia et al., 2019; Li et al., 2020). Further, we observed that the frequency of dissimilatory nitrate reduction in BS and NS was higher than denitrification, whereas the frequency of assimilatory nitrate reduction was relatively lower (**Figure 6B**; **Supplementary Table S12**). This discrepancy may be attributed to the low nitrate nitrogen concentration in BS and NS, leading to an enhanced occurrence of dissimilatory nitrate reduction and denitrification. Paddy soil has also reported a high proportion of dissimilatory nitrate reduction (Pandey et al., 2019; Pan et al., 2020).

Although the microorganisms in the sea buckthorn RN are known to perform nitrogen fixation, the specific proportions of nitrogen cycling pathways in the sea buckthorn RN microbiome have not been systematically studied. Our findings showed that nitrogen fixation represented the predominant nitrogen cycling pathway in sea buckthorn RN microbial community, accounting for an average of 47.68%. Moreover, assimilatory nitrate reduction (accounted for 22.86%) and dissimilatory nitrate reduction (accounted for 23.57%) also occupied a high proportion in RN microbial community (**Figure 6B**; **Supplementary Table S12**). *Frankia* sp. EAN1pec was the main contributor to nitrogen fixation genes in RN, accounting for 81.9%–96.9% (**Supplementary Table S13**). This indicated that *Frankia* sp. EAN1pec is a specific nitrogen-fixing microbe in sea buckthorn RN, which is completely different from the nitrogen-fixing microorganisms in previous studies on legume nodules (Xiao et al., 2017; Sharaf et al., 2019; Brown et al., 2020). Interestingly, our investigation also revealed that *Frankia* sp. EAN1pec played a role in both assimilatory and dissimilatory nitrate reduction within the RN (**Supplementary Table S13**). This could be attributed to the lower energy consumption of ammonium assimilation by plants compared to nitrate assimilation (Von Wirén et al., 2000; Ruan and Giordano, 2017). Therefore, *Frankia* sp. EAN1pec might also consume nitrate via the nitrate reduction pathway to supply a nitrogen source for plants, demonstrating the diversity and complexity of its nitrogen fixation pathway.

## 5 Conclusions

**Figure 7:**
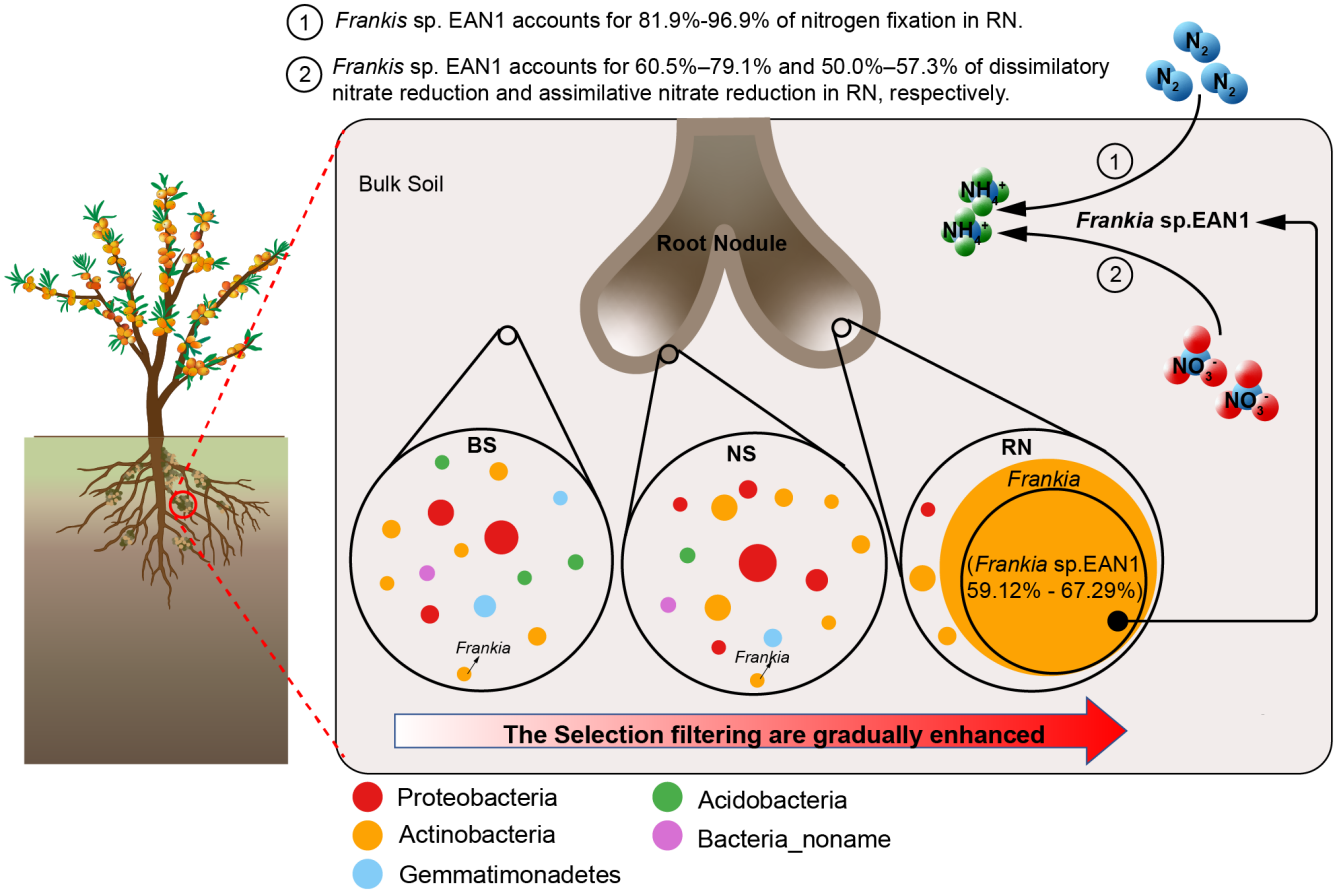
Proposed model for the relationship between sea buckthorn, soil microbes, and *Frankia* sp. EAN1. The dots represent the genus with more than 1% abundance in bulk soil (BS), nodule surface soil (NS), and nodule (RN), and the size of the dots shows the abundance of the genus.

The results demonstrate that the sea buckthorn RN has a strong selective filtering effect on soil microbes, resulting in a significant difference in microbial community composition among RN, NS, and BS samples (**Figure 7**). Additionally, there is a significant decrease in RN microbial community diversity and network complexity. Actinobacteria, specifically *Frankia* sp. EAN1pec, was the most dominant the sea buckthorn RN, and its abundance remains unaffected by changes in the eco-geographical environment within sea buckthorn growing areas. These results differ from previous research on rhizobia in legume nodule symbiosis, showing the specificity and stability of symbiotic nitrogen fixation between sea buckthorn and *Frankia* sp. EAN1pec as a result of long-term co-evolution. Furthermore, our study reveals distinct differences in the nitrogen cycle pathway composition among the RN, NS and BS microbial communities. Both NS and BS microbial communities mainly focus on dissimilatory nitrate reduction, assimilatory nitrate reduction and denitrification, while RN microbial community mainly in nitrogen fixation, with *Frankia* sp. EAN1pec being the main contributor to nitrogen fixation genes in RN. This reveals that *Frankia* sp. EAN1pec is a specific nitrogen-fixing microorganism for the nodulation of sea buckthorn. A notable observation is that *Frankia* sp. EAN1pec also plays a significant role in the nitrate reduction pathway within sea buckthorn RN, suggesting it may provide nitrogen source for sea buckthorn via nitrate reduction. This finding highlights the diversity and complexity of the nitrogen fixation pathway in *Frankia* sp. EAN1pec.

## Data availability statement

The original contributions presented in the study are publicly available. These data can be found here: https://www.ncbi.nlm.nih.gov/bioproject/PRJNA859772 and https://www.ncbi.nlm.nih.gov/bioproject/PRJNA1066471.

## Author contributions

HL: Data curation, Formal analysis, Investigation, Methodology, Visualization, Writing – original draft, Writing – review & editing, Software. BN: Data curation, Writing – original draft, Software. AD: Investigation, Writing – review & editing, Resources. CH: Supervision, Writing – review & editing. JZ: Conceptualization, Supervision, Writing – review & editing.
