# Supplementary material for: High Frankia abundance and low diversity of microbial community are associated with nodulation specificity and stability of sea buckthorn root nodule
Source: Front Plant Sci. 2024 Feb 21;15:1301447. doi: 10.3389/fpls.2024.1301447 (PMC10915256; doi:10.3389/fpls.2024.1301447)
Supplement: Supplementary file 14 [file DataSheet_1.docx]

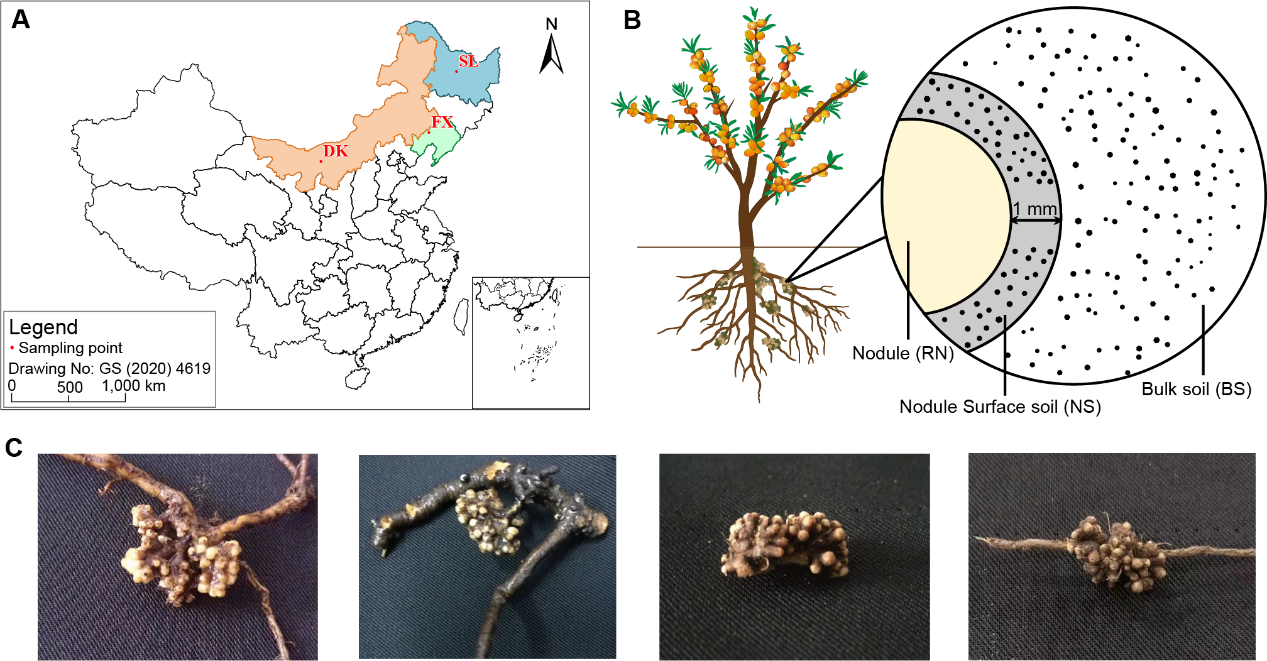


**Figure S1.** Collection samples. (A) Specific sampling sites of three sea buckthorn planting areas in this study. DK, FX, and SL are abbreviations for the three sea buckthorn planting areas, Dengkou County in Inner Mongolia Autonomous Region, Fuxin Mongolian Autonomous County in Liaoning Province, and Suiling County in Heilongjiang Province, respectively. (B) Sketch of the three compartments of samples collected from each tree, including nodule (RN), nodule surface soil (NS), and bulk soil (BS). For each biological replicate (n=3), individual plants were dug out and samples were fractionated into BS, NS, and RN compartments. Related to Figure 1 and 5. (C) Representative images of root nodules.
