# Supplementary material for: High Frankia abundance and low diversity of microbial community are associated with nodulation specificity and stability of sea buckthorn root nodule
Source: Front Plant Sci. 2024 Feb 21;15:1301447. doi: 10.3389/fpls.2024.1301447 (PMC10915256; doi:10.3389/fpls.2024.1301447)
Supplement: Supplementary file 15 [file DataSheet_2.docx]

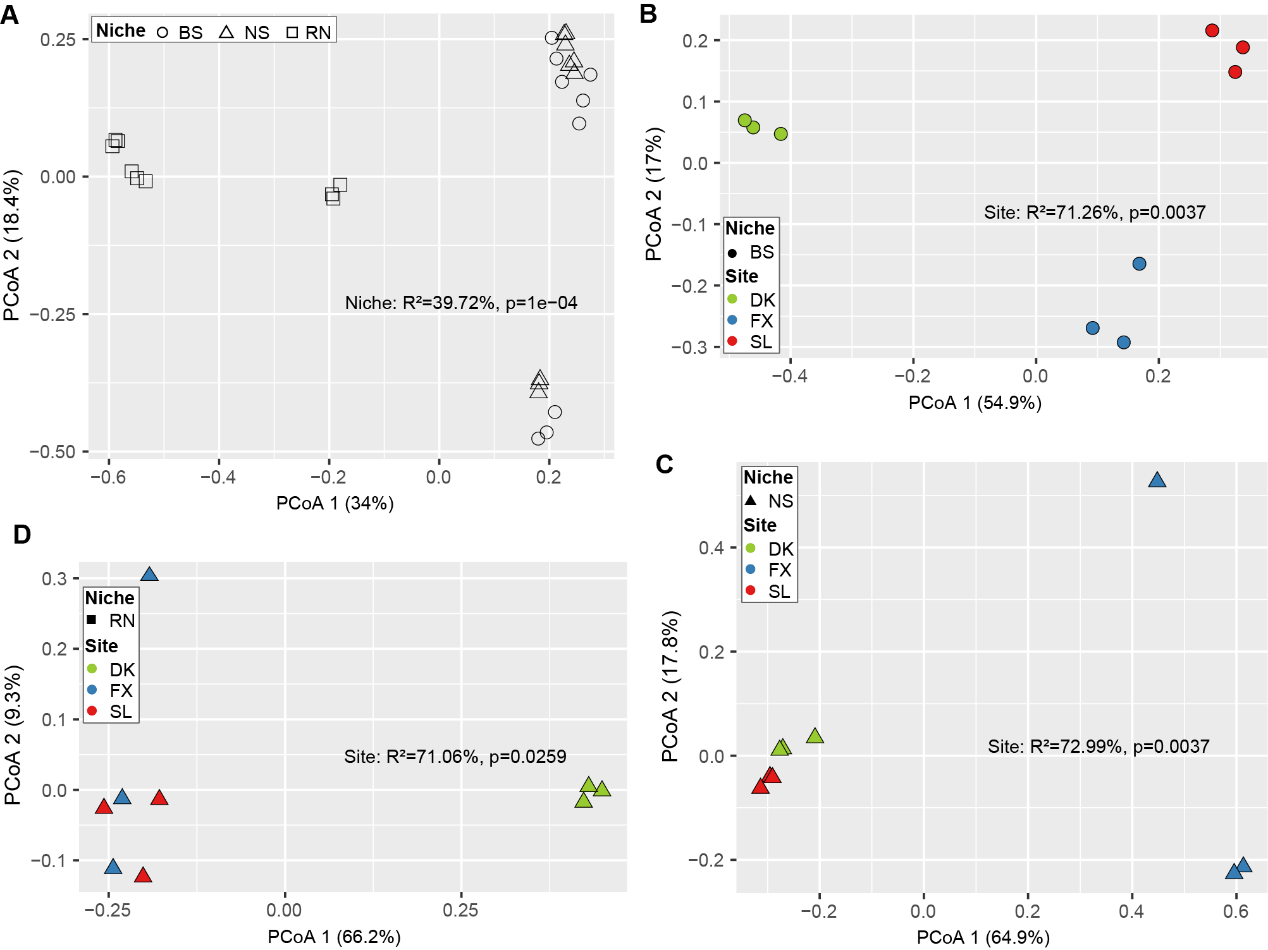


**Figure S2.** Microbial community beta diversity (based on 16S amplicon sequencing). (A) Principal coordinate analysis (PCoA) based on Bray-Curtis distance showing the microbial community composition of BS, NS, and RN in three sites. PCoA showing the impact of site on the composition of （B）BS, （C）NS, and（D）RN microbial communities, respectively. PERMANOVA was used to analyze the impact of different grouping factors on sample differences.
